# Metagenomic analysis of dental calculus in ancient Egyptian baboons

**DOI:** 10.1038/s41598-019-56074-x

**Published:** 2019-12-23

**Authors:** Claudio Ottoni, Meriam Guellil, Andrew T. Ozga, Anne C. Stone, Oliver Kersten, Barbara Bramanti, Stéphanie Porcier, Wim Van Neer

**Affiliations:** 1 Centre for Ecological and Evolutionary Synthesis (CEES), Department of Biosciences, University of Oslo, N-0316 Oslo, Norway; 2 Department of Oral and Maxillofacial Sciences, Diet and Ancient Technology Laboratory (DANTE), Sapienza University, Rome, Italy; 3 University of Tartu, Institute of Genomics, Estonian Biocentre, 51010 Tartu, Estonia; 4 Center for Evolution and Medicine, Arizona State University, Tempe, AZ USA; 5 Halmos College of Natural Sciences and Oceanography, Nova Southeastern University, Fort Lauderdale, FL USA; 6 School of Human Evolution and Social Change, Arizona State University, Tempe, AZ USA; 7 Institute of Human Origins, Arizona State University, Tempe, AZ USA; 8 Department of Biomedical and Specialty Surgical Sciences, Faculty of Medicine, Pharmacy, and Prevention, University of Ferrara, 35-441221 Ferrara, Italy; 9 Laboratoire CNRS ASM ≪ Archéologie des Sociétés Méditerranéennes (UMR 5140), Université Paul-Valéry, LabEx Archimede, F-34199 Montpellier, France; 10 Royal Belgian Institute of Natural Sciences, B-1000 Brussels, Belgium; 11 KU Leuven—University of Leuven, Department of Biology, Laboratory of Biodiversity and Evolutionary Genomics, Center of Archaeological Sciences, B-3000 Leuven, Belgium

**Keywords:** Microbial ecology, Archaeology

## Abstract

Dental calculus, or mineralized plaque, represents a record of ancient biomolecules and food residues. Recently, ancient metagenomics made it possible to unlock the wealth of microbial and dietary information of dental calculus to reconstruct oral microbiomes and lifestyle of humans from the past. Although most studies have so far focused on ancient humans, dental calculus is known to form in a wide range of animals, potentially informing on how human-animal interactions changed the animals’ oral ecology. Here, we characterise the oral microbiome of six ancient Egyptian baboons held in captivity during the late Pharaonic era (9^th^–6^th^ centuries BC) and of two historical baboons from a zoo via shotgun metagenomics. We demonstrate that these captive baboons possessed a distinctive oral microbiome when compared to ancient and modern humans, Neanderthals and a wild chimpanzee. These results may reflect the omnivorous dietary behaviour of baboons, even though health, food provisioning and other factors associated with human management, may have changed the baboons’ oral microbiome. We anticipate our study to be a starting point for more extensive studies on ancient animal oral microbiomes to examine the extent to which domestication and human management in the past affected the diet, health and lifestyle of target animals.

## Introduction

Commensal microbial communities living in or on eukaryotes play a key role in the host’s immunity, susceptibility to pathogens, metabolism and stress response^1^. The oral cavity, in particular, is an extremely rich and diverse microbial environment that can exhibit ethnic-specific variation^2^ and is a critical driver of health and disease in humans^3^. Recent metagenomic studies driven by technological advances of next-generation sequencing have demonstrated that dental calculus – plaque mineralized on the surfaces of teeth during life – from ancient specimens can be used to investigate oral microbiomes from the past^4^. Biomolecules of various origins trapped in the mineral matrix of dental calculus, originating from microbes to food residues, are a rich source of genetic and microfossil information that can inform us on health, diet and lifestyle of ancient humans and extinct hominins^5–7^.

The analytical power of dental calculus as a record of individual lifestyles from the past makes it an invaluable tool to investigate changes in the oral microbiome of ancient animals, in particular domesticated and tamed species, or species living in human-altered ecosystems. Domestication and animal management are often associated with translocations, changes in diet and feeding strategies, breeding control^8^. Unlocking the wealth of biomolecular information in animal dental calculus through paleogenomics has the potential to unravel to what extent, how and when the shifting range of human-animal relationships along (failed and successful) domestication processes influenced the diet, health and lifestyle of target animals.

Except for few studies in which historical chimpanzees have been analysed^7,9^, there is still a dearth of metagenomic studies on dental calculus in ancient animals. Current evidence is mostly based on modern oral microbiomes, showing that anthropogenic factors associated with animal management and captivity – from changes in dietary habits^10^ to life in closed environments^11,12^ – may affect the animal oral ecosystems. In some instances, cohabitation and shared environments may also lead to human-animal microbial transfer^13,14^.

In this study, we describe for the first time the oral microbiome of ancient hamadryas (*Papio hamadryas*) and olive baboons (*P. anubis*) from Pharaonic Egypt by analysing dental calculus samples. Baboons were never fully domesticated in Egypt or elsewhere, and like most primates, they are characterised by foraging behaviour and diet in their wild environment. Ancient Egyptians were renowned for their worship of animals and for keeping them as pets, from domesticated dogs and cats to wild and more ‘exotic’ animals such as baboons, gazelles, and birds^15^. Baboons and, to a lesser extent, other cercopithecine monkeys such as green monkeys (*Cercopithecus aethiops*)^16^ and macaques (*Macaca sylvanus*)^17,18^ were sacred animals and objects of a cult dedicated to Thoth, the god of writing and knowledge. More than 300 cercopithecine mummies have been found so far in three Egyptian necropoleis, Gabbanat el-Qurud, Saqqara and Tuna el Gebel^19^. The natural habitat of baboons is not an Egyptian environment, they have likely been imported from Nubia since Predynastic times, and later on from the Horn of Africa or the Arabian Peninsula^18,20^. Captivity conditions for baboons in ancient Egypt are illustrated by palaeopathological evidence of hand and foot fractures^21^ and metabolic diseases^17,22^, suggesting that the animals were subject to harsh treatment and suffered from poor health conditions.

We conducted a shotgun metagenomic analysis – the untargeted sequencing of all DNA content of a sample – of dental calculus from mummies of hamadryas and olive baboons radiocarbon dated to the end of the Third Intermediate Period and the middle of the Late Period (800–540 BC), collected at the ‘Musée des Confluences’ of Lyon (France). The specimens originated from Gabbanat el-Qurud (near Thebes, Upper Egypt), where they were held captive in unknown structures, possibly near or in temples, and buried. Initial palaeopathological analyses were conducted after the discovery of the mummies at the beginning of the 20^th^ century^19^, with a more recent examination (by WVN and SP) revealing skeletal evidence compatible with rickets, dental abnormalities (e.g. bony cysts, periodontitis), and bone lesions of unknown infectious nature on the skulls (Supplementary Table S1). We also sequenced the DNA content of dental calculus from the skeletal remains of two hamadryas baboons born and raised at the Zoological Society of Philadelphia (in Pennsylvania, USA) at the end of the 19^th^ century. We compared the oral microbiome of the ancient and historical baboons with that of a wild chimpanzee, modern, historical and prehistoric humans from the literature, in particular individuals associated with documented subsistence strategies, from hunting-gathering (including three Neanderthals) to pastoralism and farming (Supplementary Table S2). By combining metagenomic, palaeopathological and historical evidence, the overall objective of this study was to characterise the oral microbiome of baboons in conditions of captivity and reduced quality of life. Our study provides a first dataset of animal microbiota that may be used to investigate more systematically in the future the extent to which various conditions of captivity impacted the oral microbiome of non-human primates.

## Material and Methods

### Ancient Egyptian baboons laboratory procedure

All the baboon mummies were found between 1905 and 1909 in Gabbanat el-Qurud, in the so-called Valley of the Apes, near Thebes (Upper Egypt). The baboons were buried in jars, sarcophagi or wooden coffins, 1–2 meters below the ground, showing poor care in the deposition^19^. Supragingival dental calculus (Supplementary Fig. 1a) of 16 baboon mummies was collected with a scalpel at the ‘Musée des Confluences’. Sample preparation, DNA extraction, and genomic library construction for Egyptian baboons were carried out in the dedicated ancient DNA (aDNA) facilities of the Department of Biosciences of the University of Oslo. Briefly, 5–10 mg of dental calculus samples were decontaminated by rotation in bleach and UV-irradiation. DNA was extracted with silica columns using a vacuum manifold^23^ after incubation for 48 hours in an EDTA-proteinase K buffer. Double-stranded genomic libraries of ancient samples and blank controls were built as previously described^24^ with minor modifications^25^, two rounds of 10–12 cycles amplification, and sequenced to a depth ranging 15–50 million reads in one lane of a HiSeq 2500 (2 × 125 bp) (samples BB01-BB09) and in one flow cell of the NextSeq 500 (2 × 40 bp) (samples BB10-BB16) at the Norwegian Sequencing Centre core facility of the University of Oslo (Supplementary Table S3). To account for potential environmental contaminants from the depositional context and the museum storage conditions of the baboon mummies, we used sequencing data generated from two teeth of two ancient baboons (samples BB05 and BB08, Supplementary Table S1), following the same laboratory procedures as the dental calculus samples with small modifications. The two teeth showed no preservation of endogenous baboon DNA and were used as environmental controls. More details are available in the Supplementary Text.

### Historical baboons laboratory procedures

The two hamadryas baboons were born and raised at the Zoological Society of Philadelphia (in Pennsylvania, USA) at the end of the 19^th^ century. Sample preparation, DNA extraction, and shotgun build for baboon museum samples were carried out in the aDNA laboratory at the Arizona State University in Tempe, Arizona. Calculus samples (Supplementary Fig. 1b) (5–10 mg) were decontaminated using UV in a DNA crosslinker for 5 minutes, agitated, decontaminated for another 5 minutes and washed in EDTA (Ambion) on a rotating nutator for 15 minutes at room temperature. After incubation in an EDTA-proteinase K buffer, DNA was extracted using a Zymo reservoir attached to a MinElute PCR Purification kit (Qiagen) silica column. Samples and one blank control underwent a shotgun build and double-indexing amplification^24^ with slight modifications in PCR protocols from Ozga *et al*.^9^ and were sequenced on an Illumina HiSeq4000 (2 × 100 bp) lane to a depth of 10 and 22 million reads at Yale Center for Genome Analysis (YCGA). More details are available in the Supplementary Text.

### Data analysis

Raw-sequencing data were computationally processed with AdapterRemoval^26^. We removed sequence duplicates with Prinseq^27^ and performed taxonomic classification of metagenomic reads with Kraken2^28^ and a custom database of complete bacterial, viral, archaeal, mitochondrial DNA (mtDNA) and plastid genomes from the NCBI RefSeq database (https://www.ncbi.nlm.nih.gov/refseq/) masked for low-complexity regions with Dustmasker^29^. For comparative analysis, we used the custom Kraken2 database for taxonomic classification of ancient and modern dental calculus as well as modern dental plaque shotgun sequencing data from the literature (Supplementary Table S2). Kraken2 reports were combined in taxonomic read abundance tables with Recentrifuge^30^. Since we used a database of complete bacterial and archaeal genomes, we normalized the species read abundances of all the datasets, from this study and from the literature, for genome length by dividing them for the size (in Gb) reported in the NCBI RefSeq. A second normalization by total sum scaling was performed to account for library size. The *taxonomizr* library in R was used to convert NCBI taxonomic IDs to full taxonomy (from species to phylum).

We used Sourcetracker^31^, a Bayesian source-prediction tool, to estimate the proportion of reads at the genus and species level stemming from modern calculus, oral plaque, laboratory controls generated in this study, skin and soil microbiomes (see Supplementary Table S2). In the Egyptian baboon samples, we filtered out the reads stemming from laboratory and environmental controls, skin and soil (Supplementary Text). We authenticated the data by mapping the reads of the ancient baboon samples against the reference sequences of the most abundant bacterial species detected by Kraken2 after filtration (Supplementary Table S3), with BWA aln (-n 0.1, -l 1000), and we assessed the *post-mortem* damage with mapDamage^32^.

Abundance tables of Bacteria and Archaea normalized for genome length and library size were filtered to include only taxa >0.02%, and were used to calculate Bray-Curtis dissimilarities at the species, genus and family level in R, with the *vegan* library. We plotted the Bray-Curtis dissimilarities with the unweighted-pair group method with arithmetic means (UPGMA) using the *ape* library in R. Support values were estimated with the R package *pvclust*. Normalized abundance data at the phylum level were displayed with stacked bar plots using *barplot* in R. Finally, to identify bacterial and archaeal taxa that distinguish the main groups identified in the UPGMA we used LEfSe^33^, and Kruskal-Wallis test and Wilcoxon rank sum test in R with the package *dplyr*. Box plots of phyla abundance were generated with *ggpubr* in R. To further corroborate our results, we tested taxa differential abundances with DESeq2^34^ at the phylum, family and genus level.

To provide a more thorough investigation of food consumption and potential diet sources, we used Centrifuge^35^, an alignment-based metagenomic classifier that enables classification of sequences from plants, animals, fungi and many other eukaryotes, and the NCBI nt database. Centrifuge output reports were visualized with Pavian^36^.

## Results

### Quality filtering and data authentication

**Figure 1:**
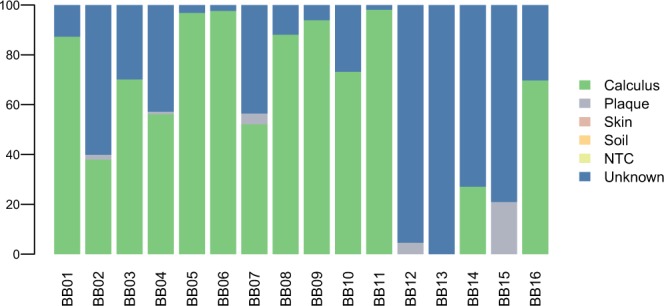
Stacked bar plot of Sourcetracker analysis showing the proportion of Kraken2 classified reads at the species level stemming from modern dental calculus, modern plaque, skin, soil and laboratory controls (NTC) in dental calculus samples of the ancient Egyptian baboons after filtration.

After quality filtering and adapters trimming, 7–21% of the reads in the ancient baboon mummies could be assigned to a taxon using the Kraken2 custom database. After removing contaminants potentially stemming from the laboratory, the depositional context, soil and skin (Supplementary Text), between 0.2 and 10.4% of the classified reads (with a maximum of 23,614 classified reads in BB11) likely represented endogenous content (see Supplementary Table S3). The results of the Sourcetracker analysis showed that before filtration the ancient baboon calculus samples contained reads mostly stemming from the laboratory controls, and that after filtration microbial DNA of oral origin ranged from 27% to 98% in 13 baboon mummies (see Fig. 1, Supplementary Fig. 2 and Supplementary Table S4). The ancient calculus samples from the literature showed high fractions of reads stemming from calculus (minimum 64% and 77% at the species and genus level in the Neanderthal from Spy), with no contributions from soil and skin microbiota, except for three historical samples (CS45, 47, 48 from Radcliffe), which showed low oral fractions (0–5%) and were removed from downstream analyses.

We only regarded baboon samples as authentic if they showed >80% of oral microbial DNA at the species level (minimum 87%, in sample BB01) and if the most abundant species showed DNA *post-mortem* damage >10% (e.g. >12% deamination rate in *Olsenella sp*. Oral Taxon 807, Supplementary Table S3). Following these criteria, the microbiome data of six of the 16 calculus samples were considered authentic and used for downstream analyses (samples BB01, BB05, BB06, BB08, BB09, BB11).

### Oral microbiome comparative analysis

**Figure 2:**
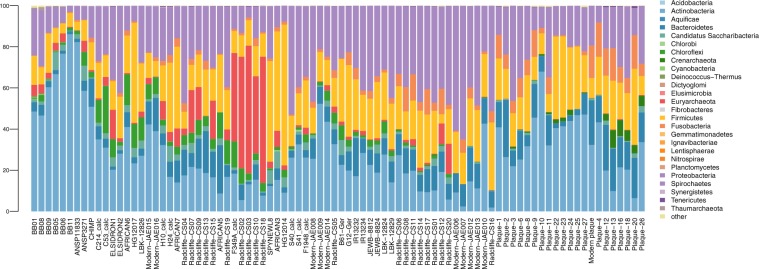
Stacked bar plot displaying relative abundance at the phylum level of Bacteria and Archaea classified with Kraken2 in ancient and modern baboons, and a selection of oral microbiomes from the literature (see Supplementary Table S2). Samples are sorted following the UPGMA clusterization.

Overall, the dental calculus of the ancient Egyptian and the two historical baboons was largely dominated by Actinobacteria, and contained minor fractions of Proteobacteria and Bacteroidetes (Fig. 2 and Supplementary Table S5). Conversely, the dental calculus of another non-human primate, a chimpanzee, showed a larger fraction of Bacteroidetes.

**Figure 3:**
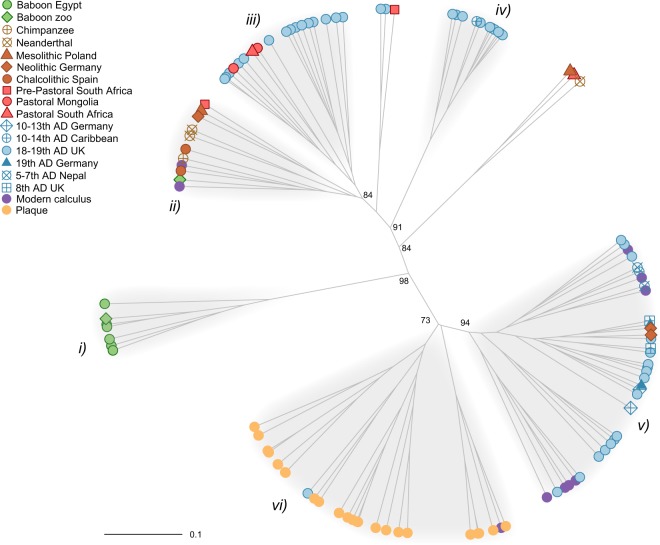
UPGMA of Bray-Curtis dissimilarities at the family level of oral microbiomes from this study and the literature (see Supplementary Table S2). AU *p*-values computed by multiscale bootstrap resampling in *pvclust* are indicated for the main clusters.

The overall topology of the UPGMA clustering of Bray-Curtis dissimilarities at the family level (Fig. 3, Supplementary Table 6) showed at the lower end of the dendrogram that the microbiome of dental plaque samples is distinct from that of modern and ancient calculus samples, as recently observed^37^. Most of the calculus samples from modern humans (eight out of ten) clustered with historical human calculus samples from the UK, Germany, Nepal and Guadeloupe, and two Neolithic samples from Germany. All the remaining ancient human samples, clustered on the upper side of the dendrogram. The six ancient Egyptian baboon mummies and one historical baboon sample, ANSP1183, represented a separate group. Interestingly, the wild chimpanzee and individuals characterised by hunter-gatherer and pastoral lifestyle (two Neanderthals from El Sidron, two individuals from Chalcolithic Spain, one from Pre-Pastoral South Africa, one from Mesolithic Poland, see also Supplementary Table S2 and Supplementary Text) clustered in the same group together with the second historical baboon sample (ANSP3271). In this cluster, we also found one Neolithic farmer from Germany and two modern calculus samples.

The UPGMA of Bray-Curtis dissimilarities at the genus and species level confirmed the overall structure observed at the family level by supporting the clustering of the ancient Egyptian baboons in a distinct group together with the historic sample ANSP1183. Minor changes in the positioning of some samples at the genus level (ANSP3271 and African6) (Supplementary Fig. 3) may be due to higher inaccuracy of classification at lower taxonomic ranks, and the presence of species not yet classified on a higher taxonomic level (e.g. *Anaerolineaceae* bacterium oral taxon 439, which lacks a genus definition).

We focused our comparative analysis on six highly supported clusters identified in the UPGMA, (*i*) the ancient baboons, (*ii*) the cluster encompassing the wild chimpanzee, hunter-gatherers (including two Neanderthals) and some pastoralists, (*iii*) a cluster of historical samples from the UK and three pastoralists from Mongolia and South Africa, (*iv*) a cluster of historical samples from the UK and Guadeloupe, (*v*) mixed historical and modern calculus samples, including two European Neolithic farmers, and (*vi*) dental plaque from modern humans. The LefSe analysis conducted on bacterial and archaeal taxa from phylum to family level revealed that the Actinobacteria phylum, and Atopobiaceae family including the genus *Olsenella* were the main discriminant taxa of the baboon group (clade *i*) (Supplementary Fig. 4, Table S6). The group comprised of the wild chimp, hunter-gatherers and pastoralists, which also includes the second historical captive baboon (clade *ii*), was characterised by bacteria of the Chloroflexi phylum, in particular the Anaerolineaceae family, and Actinomycetaceae bacteria. The two clusters of historical calculus samples, clades *iii* and *iv*, were dominated by Bacteroidetes, and Archaea bacteria of the phylum Euryarchaeota, in particular Methanobacteriaceae, respectively. Proteobacteria characterized clade *v*, which contained mostly modern and historical calculus, whereas modern dental plaque samples (clade *vi*) were marked by bacteria of the phyla Actinobacteria, Firmicutes and Fusobacteria. The Kruskal-Wallis test and the Wilcoxon rank sum test performed on the bacterial phyla abundances confirmed that the baboons had significantly higher frequencies of Actinobacteria (Wilcoxon rank sum test p < 0.01, Supplementary Fig. 5, Table S7), and lower frequencies of Firmicutes and Fusobacteria (p < 0.1) than the other groups. Chloroflexi were significantly more abundant in clade *ii* of the wild chimpanzee, hunter-gatherers and pastoralists (p < 0.05), and less abundant in the modern dental plaque samples (clade *vi*), while Proteobacteria marked clade *v*, the historical and modern calculus samples (p < 0.01).

Overall, the DESeq2 analysis confirmed the reported taxonomic differences at the phylum and family level when comparing the baboons against the other clades (Supplementary Tables S8–S10, Fig. S6). At the genus level, we confirmed that *Olsenella* discriminated the baboons from all the other groups of oral microbiota.

### Commensal and pathogenic oral species in the ancient baboons

For the six ancient Egyptian and the two historical baboon calculus samples, the most abundant microbial species were *Olsenella* sp. Oral Taxon 807 and *Actinomyces* sp. Oral Taxon 414. Extremely low preservation of endogenous baboon DNA was observed in both the ancient and historical samples (<10 reads mapping to the reference *P. hamadryas* and *P. anubis* mtDNA). In five of six baboon mummies, we found small numbers of reads assigned to the red complex pathogens *Tannerella forsythia*, *Porphyromonas gingivalis* and *Treponema denticola* (0.2–0.8% in total), whereas higher proportions were found in the two historical baboons (1.3 and 4%) and in the Egyptian sample BB08 (3.6%) (Supplementary Table S11). Among other putative oral pathogens that were found in the dental calculus of the ancient Egyptian baboons, *Rothia mucilaginosa* possessed the highest frequencies (up to 1.6% in BB08). Additionally, the ancient baboons showed higher proportions of *Streptococcus mitis*, *Stretococcus mutans* and *Veillonella parvula* than the two historical specimens.

To explore the origin of the bone lesions observed on the baboon skulls in more depth, we investigated whether they could have been caused by actinomycotic infection, which is commonly associated with lesions in the oral cavity, the temporal bone and the skull base in humans^38^. We made a competitive alignment of the reads against available reference data (complete genomes, contigs and scaffolds) of 34 *Actinomyces* species deposited in GenBank. In the two historical baboons *A. dentalis* was the most represented species (76–80%), whereas in the ancient Egyptian mummies it ranged between 17–82% (Supplementary Table S12). Two *Actinomyces* species, *A. israelii* and *A. gerencseriae*, are known to be responsible for about 70% orocervicofacial infections in humans^39^. While we found only low frequencies of *A. israelii* (0.9–2.6%) in all the samples, *A. gerencseriae* reached an abundance of 42% in one baboon mummy (samples BB11), whereas abundances ranged from 1–24% in the other samples. The results were corroborated by the presence of *post-mortem* damage in DNA molecules aligned to *A. gerencseriae* ranging between 10–21% in the ancient baboons.

The analysis with Kraken2 additionally revealed the presence of a human oral commensal, *Methanobrevibacter oralis*, in one historical baboon (ANSP3271) and in one Egyptian mummy (99 reads in BB06, the others showing <20 reads) (Supplementary Table S13). To further corroborate the presence of this Archaea species in the oral microbiome of the ancient baboon BB06, we aligned the reads against the reference genome (230 reads in total) and detected *post-mortem* damage typical of aDNA molecules (deamination >25% at the 5′- and 3′-ends). The results were further supported by a competitive alignment against 13 *Methanobrevibacter* genomes deposited in GenBank, confirming that the majority of the reads in the historical and in the mummified baboon BB06 sample (53% and 38% respectively) aligned to *M. oralis*.

### Dietary analysis with Centrifuge

Results of the Centrifuge analysis were mostly characterised by spurious classifications of reads to plant and animal taxa, as they were widely represented in the negative controls. At the family level, we observed the highest number of hits with Poaceae (grasses) and Fabaceae (legumes, peas and beans) taxa. After excluding all potential spurious hits represented by >10 reads in the negative controls, both the Centrifuge and the Kraken2 analysis detected cucumber (*Cucumis sativus*, 259 and 51 reads respectively) and watermelon (*Citrullus lanatus*, 24 and 11 reads respectively) in sample BB11. As further means of verification, we used BLASTn to classify the reads assigned to cucumber by Kraken2 (purportedly matching mtDNA and/or plastid reference sequences) against the NCBI nt database, and found unique hits to a *Cucumis* species in 51 of 59 reads (86%), with *Cucumis sativus* as first best hit in all the instances (scores ranging 63–126). The same analysis on watermelon reads showed unique hits to *Citrullus lanatus* in seven of the 11 reads (64%) classified by Kraken2 (scores ranging 76–122). After aligning the shotgun reads against the mtDNA reference sequence of *Cucumis sativus* and *Citrullus lanatus*, the *post-mortem* damage analysis showed very low level of cytosine deamination for cucumber (<2%, 4045 reads mapped), whereas for watermelon damage patterns could not be assessed due to the low number of reads aligned (53).

## Discussion

### DNA preservation in dental calculus of ancient Egyptian baboons

Recent studies have demonstrated that the mineral matrix and high microbial cell density (estimated at more than 200 million cells per milligram) of dental calculus make it an optimal environment for DNA preservation in archaeological material^4–6^. We could detect oral bacterial species (e.g. *Olsenella* sp. Oral Taxon 807, *Actinomyces* sp. Oral Taxon 414), in all the ancient Egyptian baboon mummies that we analysed. However, based on the authentication and the filtration strategies that we followed, we were able to successfully investigate the oral microbiome of six of the 16 ancient Egyptian baboon specimens analysed. Eleven samples showed large proportions of exogenous reads reported by the Sourcetracker analysis at the species level as unknown (Supplementary Table S4, Fig. S2).

We also noticed a large fraction of reads of environmental origin most likely associated with the depositional context (Supplementary Text). Most notably, more than 4,000 reads were assigned to Halobacteria, halophile Archaea that require high salt concentration to grow, in sample BB02. It should be noted that halophile archaeal and bacterial species, assessed as non-authentic due to the lack of significant *post-mortem* damage (<2%), were also found in the teeth used as environmental post-depositional control. We believe this microbial component may stem from peculiar salt deposits and concretions that are often found in burial chambers of archaeological sites of Egypt and the Nile Valley (Supplementary Fig. 6), where salt crystals may grow upon archaeological remains.

The proportion of endogenous baboon DNA appeared to be extremely low in the dental calculus of the Egyptian mummies and the two historical zoo specimens investigated. This is not surprising given the relatively low abundance of host DNA in archaeological dental calculus and the inefficiency of shotgun sequencing to obtain it in significant fractions^6^. For this reason, targeted-enrichment is usually necessary in any prehistoric or historic populations^40^. Overall, our data prove that archaeological dental calculus is a rich source of endogenous microbial DNA even in extremely poor environments for biomolecular preservation such as arid ancient Egypt and after long-time museum storage (more than a century).

### The oral microbiome of ancient Egyptian baboons in captivity

Baboons are not native to the country of Egypt. The current distribution of *Papio* species spans much of sub-Saharan Africa and the hamadryas baboon is the only species found outside the African range, in the southeast coast of the Arabian Peninsula, where it most likely dispersed during the Late Pleistocene through the Bab-el-Mandab strait^41^. Ancient Egyptians and Nubians had a major role in the translocation of baboons to Egypt^16,18^. Written and iconographic evidence dating from the Old Kingdom until the Hellenistic period (4,500 to 2,000 years ago) revealed that they undertook expeditions south of the Nile Valley and to the yet undiscovered “Land of Punt”, most likely located in East Africa^42,43^ or in the Arabian Peninsula^44^. This place was a trade centre for valuable goods and exotic plants and animals, including baboons, which were brought to Egypt, kept as pets and associated with the cult of Thoth^20^.

In agreement with previous studies^37^, our analysis showed that human dental plaque (clade *vi*) and calculus represent two distinct oral microbiomes. All the baboon mummies and one historical baboon (ANSP11833) born and raised in a zoo in the United States clustered together and possessed a distinct oral microbiome (clade *i*). Interestingly, the clustering of baboon samples analysed in different laboratories (see Supplementary Text), from two completely different contexts and taphonomic conditions (Egyptian tombs *vs* a zoo) and age (about 2,500 years *vs* 100 year) suggests that our results are unlikely to be biased by diagenetic factors or laboratory methods. However, differential species preservation and potential taxonomic loss are as yet not fully understood^6^, and only the systematic analysis of more samples representing various taphonomic conditions may help in the future to address this issue.

The second historical baboon (ANSP3271), born and raised in that same zoo as ANSP11833, clustered with a wild chimpanzee and other specimens characterised by hunting-gathering (including Neanderthal) and pastoral strategies (clade *ii*). The low number of samples analysed in this study and the lack of wild baboon samples prevent us from making generalizations about the impact of captivity on the animal oral microbiome. However, based on the evidence available, different scenarios may be envisaged. Throughout their natural semi-desert range, baboons feed mostly on leaves, fruits and shoots, and in conditions of habitat degradation they may rely on invertebrates and small mammals, as well as agricultural crops and refuse from human environments^45,46^. On the one hand, the overall clustering of the baboons’ oral microbiomes may be representative of their omnivorous dietary habits and opportunistic feeding strategy in the wild, which makes them adaptive to a wide range of food sources, including nutritional management by humans. On the other hand, more complex scenarios may suggest that one baboon (ANS3271) clustering in clade *ii* with a wild non-human primate (chimpanzee) and hunter-gatherers retained a wild foraging-like microbiome signature, while the rest of the baboon samples diverged from natural conditions due to captivity and life in a confined anthropogenic environment. In fact, despite their adaptability, baboons are strongly dependent on natural resources for proteins, minerals, vitamins and even medical chemicals^45^. Furthermore, it has been observed that traditional nutritional management of captive groups in zoos based on high-quality food provisioning, a diet that is low in fiber and rich in simple sugars (e.g. fruit), leads to food-based dominance, increased aggressiveness and immunodepression, resulting in dental disease and microbiome dysbiosis^47,48^. Similar factors may have come into play in ancient Egypt, causing a disruption of baboons’ social patterns and overall health depression.

Palaeopathological analyses revealed dental and skull abnormalities in the baboons from Gabbanat el-Qurud, suggesting poor health conditions. While red-complex bacteria were observed at low abundances in the ancient baboons, we detected higher frequencies of other pathogenic species such as *R. mucilaginosa*, *S. mitis*, *S. mutans* and *V. parvula* (Supplementary Table S8). Species of the red-complex act as ‘late colonizers’ in biofilm maturation through time and are often abundant in archaeological human dental calculus, which represent fully mature biofilm profiles^37^ (Supplementary Table S11). The low abundance of red-complex bacteria in the ancient baboons may be the result of differential pattern of biofilm maturation, even though, in some instances, a confident abundance estimate was hampered by the low number of reads found (<30 for some ancient samples).

Poor health conditions of the ancient Egyptian baboons are suggested by the analysis of oral actinomycetes, which showed that four of the six ancient baboons had higher proportions (19–42%) of *A. generencseriae*, one of the facultative pathogenic species responsible of actinomycosis^49^, compared to the historical baboons and modern humans (0–13%). This suggests that the Egyptian baboons may have suffered from different degrees of actinomycotic infections, which may have caused bone lesions on the skull of one individual (BB08).

In at least one of the baboon mummies (BB06) and in one modern baboon (ANSP3271), we detected *M. oralis*, an archaeal human commensal. This microbe was not detected in the wild chimpanzee sample, but was recently also detected in the oral microbiome of Neanderthals^7^. In ancient Egypt, baboons were probably captured at an early age, raised and fed in confinement conditions in human environments (households and/or temples), where they may have acquired *M. oralis* through e.g. food-borne horizontal transmission. We also cannot exclude that the ancient baboons were born in captivity. In fact, Egyptians were renowned for their attempts to reproduce and rear animals in captivity^15^, a situation which may have likely resulted in vertical transmission of human commensal microbial species. However, whether this archaeal species occurs naturally in the oral baboon microbiome remains contentious, as shared bacterial species between mammal species may not necessarily imply direct interaction between them^50^. More extensive analysis of oral ecology in non-human primates may help to fill this knowledge gap in the future.

### Diet in ancient Egyptian baboons

Shotgun metagenomic datasets have recently been used for ancient dietary analyses, but are often problematic due to spurious hits causing false positive signals, and as a result of limitations and assumptions associated with the use of modern reference sequences that may not reflect ancient taxa^50^. The analysis of plant taxa conducted on our shotgun dataset mostly returned hits to the families Fabaceae (legumes, peas, beans) and Poaceae (grasses), but in most instances no further investigation at deeper taxonomic levels was possible due to the concurrent presence of spurious hits in the negative controls. The analysis with the custom database in Kraken2, the nt database in Centrifuge, and NCBI nt Blast, detected the presence of cucumber (*Cucumis sativus*) and watermelon (*Citrullus lanatus*). The low level of deamination of the DNA molecules mapping to cucumber suggests that they may originate from some unknown source of contamination. Cucumber is native to the Indian subcontinent, and current linguistic and iconographic evidence support its introduction in Africa in Medieval times^51^. Watermelon, on the other hand, is native to Africa, and iconography and seeds found in contexts as old as more than 4,000 years point to Northeast Africa as the original centre of domestication^52^. However, the damage patterns could not be assessed due to the limited number of hits, and we cannot exclude that the reads assigned to watermelon belong to different ancient species not yet represented in the databases. We stress that extreme caution is needed when inferring food sources from shotgun reads, in particular without authentication strategies or other sources of evidence. We suggest that future dietary analysis might have more success through targeted-enrichment methods and by combining evidence from microfossil and residue analysis.

## Conclusions

This study demonstrates that dental calculus is a powerful tool to reconstruct oral microbiomes of animals from the past. Here, we characterised the oral microbiome of ancient Egyptian and historical baboons in captivity, demonstrating that they possessed a distinctive microbial ecosystem largely dominated by Actinobacteria. While this could reflect the omnivorous diet and the flexible foraging lifestyle of baboons in the wild, we cannot exclude that various factors associated with the condition of captivity affected the baboons’ oral microbiome. Far away from the natural semi-arid conditions which they were adapted to, the ancient Egyptian baboons suffered from poor health, which may have been caused by life in the confined environment of temples and human nutritional management.

However, we stress that multiple factors may have shaped the oral microbiome of the baboons in captivity, from health and diet, to host genome, interaction with humans or other unknown factors. Our study represents a first dataset of non-human primates oral microbiomes in captivity, and future analyses of dental calculus of archaeological and modern wild baboons, as well as other primates, will help to gain a more thorough understanding of the effects of captivity conditions on the animal oral ecology.

## Supplementary information

Supplementary text and figures

Supplementary Dataset

## Data Availability

The sequencing data generated in this study are available in the European Nucleotide Archive repository, with the study accession number PRJEB34875. Supplementary data that support the findings of this study are available in the journal’s website.
