# Supplementary material for: Metagenomic analysis of dental calculus in ancient Egyptian baboons
Source: Sci Rep. 2019 Dec 23;9:19637. doi: 10.1038/s41598-019-56074-x (PMC6927955; doi:10.1038/s41598-019-56074-x)
Supplement: Supplementary file 1 — Supplementary text and figures [file 41598_2019_56074_MOESM1_ESM.pdf]

Metagenomic analysis of dental calculus in ancient Egyptian baboons

Claudio Ottoni, Meriam Guellil, Andrew T. Ozga, Anne C. Stone, Oliver Kersten, Barbara Bramanti, Stéphanie Porcier, Wim Van Neer.

Electronic supplementary material

Ancient Egyptian baboons

For all mummies, wrappings were not preserved and only the skull and limited post-cranial elements were available for analysis. 5-10 mg of dental calculus samples (Supplementary Figure 1a) were first decontaminated by rotation in 4% bleach for up to 15 minutes, rinsed in nuclease free water, dried with ethanol and UV-irradiated for 15 minutes in a crosslinker. Two lysis protocols were used; in the first, samples were incubated in 1mL of EDTA 0.5M pH 8 at room temperature. After 24 hours we added proteinase K (20mg/mL) and N-Lauryl sarcosine, and incubated the samples at 55°C for 24 hours, refreshing the proteinase K after six hours. In a second phase of the project, samples were digested in an EDTA 0.5 M-proteinase K (20mg/mL) buffer for 48 hours at 55°C on a nutator. After lysis, DNA was extracted with silica columns[1] using a vacuum manifold. The two tooth samples (from mummies BB05 and BB08, Supplementary Table 1) were UV-irradiated in a crosslinker for 10 minutes on each side and wiped with 4% bleach. Root fragments were pulverized with a mortar and incubated in EDTA-proteinase K buffer for 24 hours. Double-stranded genomic libraries were built as previously described[2], with minor modifications[3]. Thirty uL of genomic libraries were split in two or three single-indexing amplification reactions of 12 cycles (50uL volume with 2.5U of AmpliTaq Gold, 3mM $MgCl_2$, 0.4 mg/mL BSA, 200 uM each dNTP, 200 nM of primer IS4 and index primer) purified with magnetic beads, reamplified for 10-12 cycles with IS5/IS6 primers, and size-selected with a BluePippin (2% Agarose 100-600bp, Sage Science). Extraction, library and amplification controls were processed in parallel with the samples. All sample libraries were pooled in equimolar concentrations and shotgun sequenced in paired-end mode in one lane of a HiSeq 2500 (125 bp) and in one flow cell of the NextSeq 500 (40 bp) at the Norwegian Sequencing Centre core facility to obtain a final sequencing depth of about 15-25 million reads per sample. Two aliquots of the libraries of the samples BB03, BB05, BB06 were sequenced in two different batches to obtain higher coverage for some oral bacterial species.

Historical baboons laboratory procedures

The two hamadryas baboons were born and raised at the Zoological Society of Philadelphia (in Pennsylvania, USA) at the end of the 19th century. No information is available about kinship of the two baboons. The laboratory at the Arizona State University in Tempe is a UV-equipped, class 10,000 HEPA filtration cleanroom and researchers are required to wear full length sterile suits, hairnets, facemasks, and eye protection throughout experiments. Calculus samples (Supplementary Figure 1b) (5-10 mg) were decontaminated for 5 minutes using UV in a DNA crosslinker, agitated, and decontaminated for another 5 minutes. Samples were transferred to a 2 mL tube and washed with 1 mL of 0.5M EDTA (Ambion) on a rotating nutator for 15 minutes at room temperature. Samples were centrifuged at maximum speed for 3 minutes and the supernatant was removed and discarded. A total 1 mL of fresh 0.5M EDTA and 100 mL of proteinase K (Qiagen) were added to the pellet and rotated for 8 hours at 37°C followed by an overnight rotating nutator incubation. Samples were centrifuged at maximum speed for 3 minutes and the supernatant was added with 12 mL of PB Buffer (Qiagen) to a Zymo reservoir attached to a MinElute PCR Purification kit (Qiagen) silica column (with a 50 mL falcon tube in order to catch flowthrough). Samples were spun at 6k rpm for 4 minutes, rotated 180° and spun another 2 minutes. The MinElute column was transferred to a new collection tube and purified according to manufacturer specifications, ending with a final elution of 30 µL. Samples underwent a shotgun build and double-indexing amplification[2] with slight modifications in PCR protocols from Ozga et al. (2019)[4]. Concentrations were checked using a DNA1000 Bioanalyzer chip (Agilent) and sequenced on an Illumina HiSeq 4000 2x100bp lane at Yale Center for Genome Analysis (YCGA).

Data analysis and reads filtering

Raw-sequencing data were computationally processed with AdapterRemoval[5] for trimming adapter sequences, reads quality filtering (--minlength 30 --minquality 15 --trimns –trimqualities), and merging paired reads. Sequence duplicates were removed with Prinseq[6]. For taxonomic classification of metagenomic reads from this study as well as the literature (Supplementary Table S2), we used Kraken2[7], which performs accurate classification using exact alignment of k-mers against a customizable reference database. We built a custom database of k-mers from all complete bacterial, viral, and archaeal genomes downloaded from the NCBI RefSeq database (https://www.ncbi.nlm.nih.gov/refseq/). For the Archaea, we also added partial assembled genomic data, to include species for which complete genomes are not yet available in RefSeq, such as *Methanobrevibacter oralis*. To provide an indication of potential food consumption, we included mitochondrial DNA (mtDNA) and plastid genomes from the NCBI RefSeq in the database. All genomes were masked in Kraken2 for low-complexity regions with Dustmasker[8], to reduce the impact of potential spurious classifications.

We used Recentrifuge[9] to merge Kraken2 reports in species abundance tables. We downloaded genome information for prokaryotes organisms (https://www.ncbi.nlm.nih.gov/genome/browse#!/prokaryotes/) and used the genome length data (in Gb) to normalize reads abundances with a custom python script. A second normalization by total sum scaling was performed to account for library size.

We used Sourcetracker[10], a Bayesian source-prediction tool, to estimate the proportion of reads stemming from various microbiota. The Sourcetracker analysis was performed on normalised bacterial taxa reads abundance at the genus and species level using the metagenomic datasets of modern human dental calculus[11], oral plaque, skin and soil from the European Nucleotide Archive (ENA) (https://www.ebi.ac.uk/ena), and the laboratory control generated in this study as sources, whereas all the ancient samples were used as sink (see Supplementary Table S4) .

All identified species with >10 reads in the negative controls and >200 in the ancient teeth environmental controls were removed from the Kraken2 reports of the ancient Egyptian baboons. We adopted these less stringent filtering values to avoid removing authentic oral species that were found at low level in the negative laboratory control (e.g. *Olsenella sp.* Oral Taxon 807, 6 reads) and in the Egyptian baboon teeth samples (e.g. *Actinomyces oris*, 130 reads), where oral bacterial species may be retrieved[12]. By using the Egyptian baboon teeth samples as environmental post-depositional controls, we could detect and filter out halophile Archaeal (Halobacteria) and bacterial species (e.g. *Nocardopsis dassonvillei*) that were assessed as non-authentic due to the lack of significant *post-mortem* damage (<2%) from the archaeological samples. Given the high fraction of endogenous oral content in the historic baboons ANSP3271 and ANSP11833 (ranging 92-99%, Supplementary Table 4), no reads filtering was performed on these samples. Based on the Kraken2 results, we also generated a list of species identified in the soil and skin ENA dataset, and, taking a more conservative approach, removed all species described in the Human Oral Microbiome Database (HOMD) from this list. The resulting list was used to filter out soil and skin contaminant species from the ancient Egytpian baboon samples. Both filtered and unfiltered abundance data of the Egyptian baboons were analysed with Sourcetracker (Supplementary Table S4, Supplementary Figure 2).

The *taxonomizr* library in R was used to retrieve full taxonomic data of species abundances (from species up to phylum) and generate genus, family and phylum abundances, which were used for downstream comparative analyses (UPGMA, DESeq2, LEfSe). In the UPGMA we identified the clusters to use for comparative analysis based on statistical support (AU *p*-values >70) and numerosity (n>5).

Metadata collection

Given the strong influence of individual factors, from lifestyle to diet and health, on oral microbiome compositions, metadata collection is key for interpreting differences in bacterial communities. In this study, we retrieved additional information concerning subsistence strategies and health of the individuals used in the comparative analysis from the literature, as reported in Supplementary Table S2. In particular, carbon and

nitrogen stable isotopes of the samples from Guadeloupe[13] suggested a mixed diet of terrestrial and marine resources (though the isotopic signatures for one sample are missing). Strontium isotope analysis of ancient humans from the Chalcolithic site of Camino del Molino, in Spain[14], indicated transhumant pastoralism as an important economic practice in the region. Two individuals from the site of Samdzong in Nepal were regarded in a recent study as agriculturalists cultivating barley and buckwheat[15]. We believe that complementary dataset integrating evidence from archaeological contexts, history, stable isotopes, proteomic and paleopathological analysis may help in the future to define individual lifestyles in association with oral microbiomes, and to provide a better understanding of affinities and divergences among individual oral microbiota.

References

1. Dabney, J. *et al.* Complete mitochondrial genome sequence of a Middle Pleistocene cave bear reconstructed from ultrashort DNA fragments. *Proc. Natl. Acad. Sci. U. S. A.* **110**, 15758–15763 (2013).
2. Meyer, M. & Kircher, M. Illumina sequencing library preparation for highly multiplexed target capture and sequencing. *Cold Spring Harb. Protoc.* **2010**, db.prot5448 (2010).
3. Namouchi, A. *et al.* Integrative approach using Yersinia pestis genomes to revisit the historical landscape of plague during the Medieval Period. *Proc. Natl. Acad. Sci. U. S. A.* 201812865 (2018).
4. Ozga, A. T. *et al.* Oral microbiome diversity in chimpanzees from Gombe National Park. *Sci. Rep.* **9**, 17354 (2019).
5. Schubert, M., Lindgreen, S. & Orlando, L. AdapterRemoval v2: rapid adapter trimming, identification, and read merging. *BMC Res. Notes* **9**, 88 (2016).
6. Schmieder, R. & Edwards, R. Quality control and preprocessing of metagenomic datasets. *Bioinformatics* **27**, 863–864 (2011).
7. Wood, D. E. & Salzberg, S. L. Kraken: ultrafast metagenomic sequence classification using exact alignments. *Genome Biol.* **15**, R46 (2014).
8. Morgulis, A., Gertz, E. M., Schäffer, A. A. & Agarwala, R. A fast and symmetric DUST implementation to mask low-complexity DNA sequences. *J. Comput. Biol.* **13**, 1028–1040 (2006).
9. Martí, J. M. Recentrifuge: Robust comparative analysis and contamination removal for metagenomics. *PLoS Comput. Biol.* **15**, e1006967 (2019).
10. Knights, D. *et al.* Bayesian community-wide culture-independent microbial source tracking. *Nat. Methods* **8**, 761–763 (2011).
11. Velsko, I. M. *et al.* Microbial differences between dental plaque and historic dental calculus are related to oral biofilm maturation stage. *Microbiome* **7**, 102 (2019).
12. Mann, A. E. *et al.* Differential preservation of endogenous human and microbial DNA in dental calculus and dentin. *Sci. Rep.* **8**, 9822 (2018).
13. Laffoon, J. E. & de Vos, B. Diverse origins, similar diets: an integrated isotopic perspective from Anse à la Gourde, Guadeloupe. *Communities in Contact: Essays in Archaeology* (2011).
14. Merner, C. Reconstructing Southeast Iberian copper age mobility: a strontium isotope analysis of the Camino del Molino mass burial. (Memorial University of Newfoundland, 2017).
15. Schmidt, C. W., Beach, J. J., McKinley, J. I. & Eng, J. T. Distinguishing dietary indicators of pastoralists and agriculturists via dental microwear texture analysis. *Surf. Topogr.: Metrol. Prop.* **4**, 014008 (2015).

a)

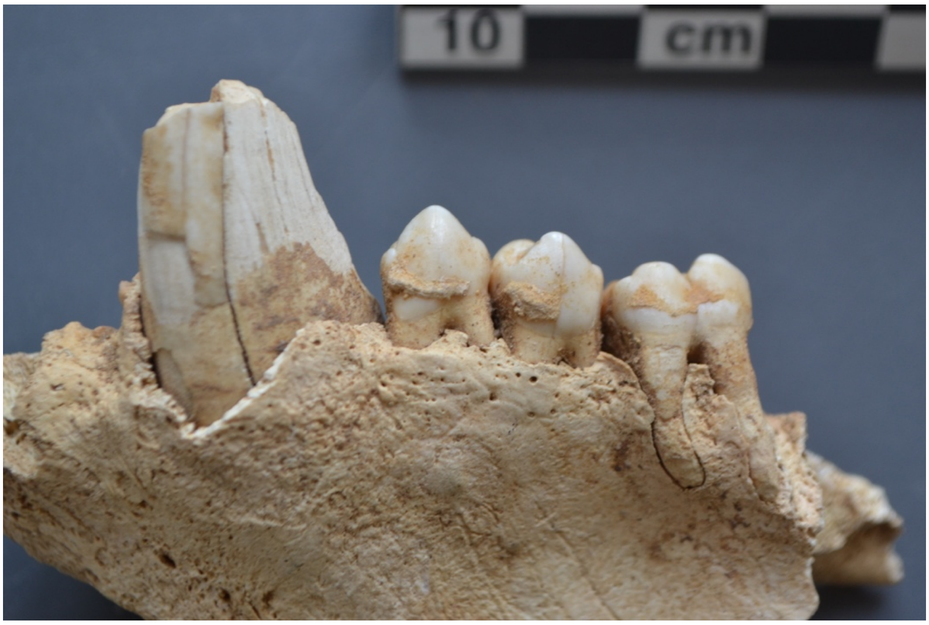

b)

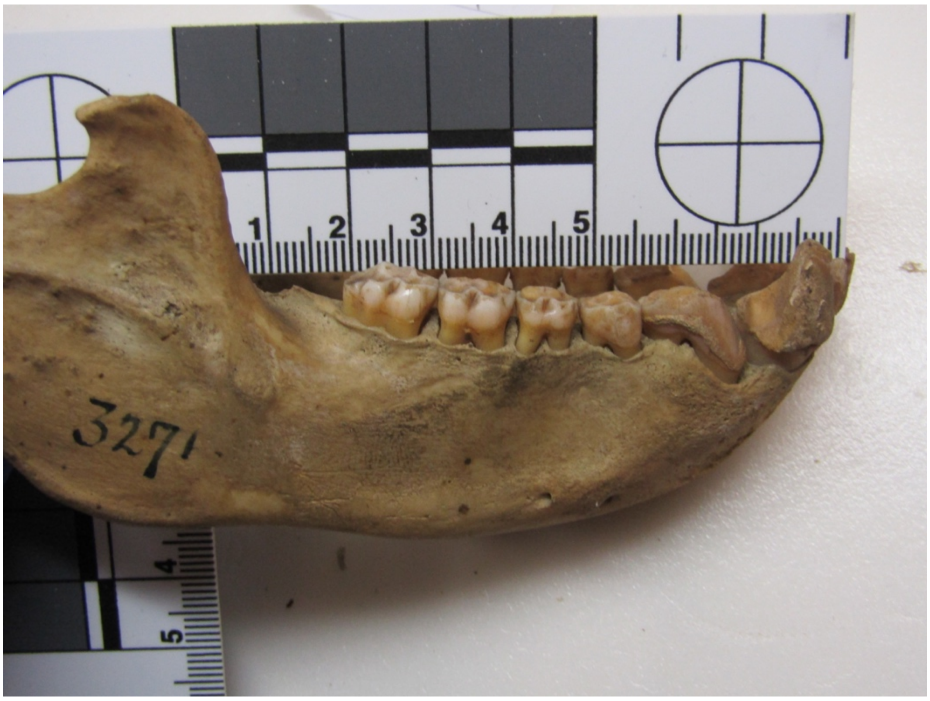

Supplementary figure 1. Two dental calculus samples analysed in this study, (a) ancient Egyptian baboon, and (b) historical baboon.

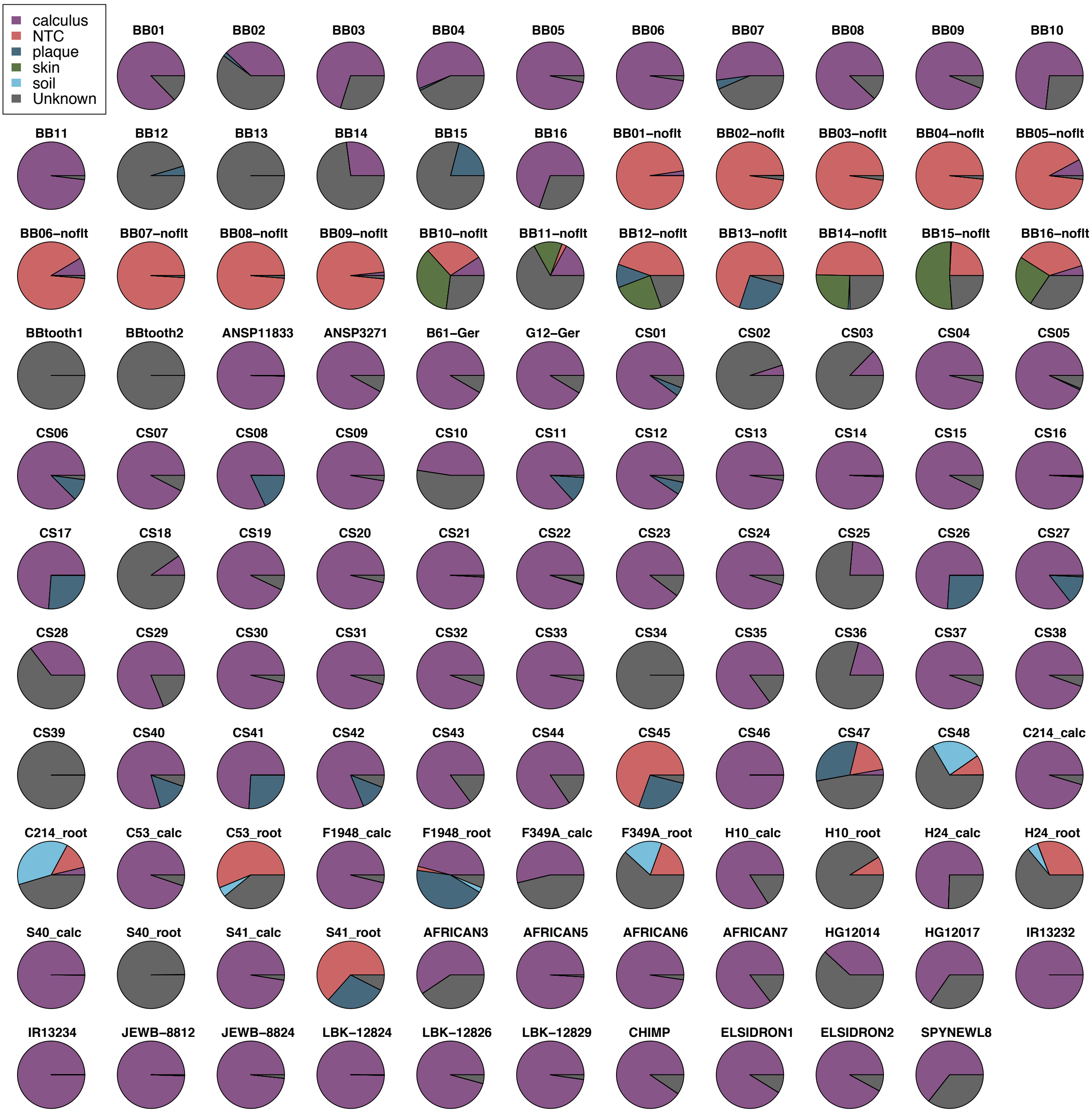

Supplementary figure 2. Pie charts representing the proportion of Kraken2 classified reads stemming from modern dental calculus, modern plaque, skin, soil and laboratory controls (NTC) (Supplementary Table S4) in calculus samples of the baboons analysed in this study and of ancient samples from the literature (Supplementary Table S2) estimated with Sourcetracker at the species level.

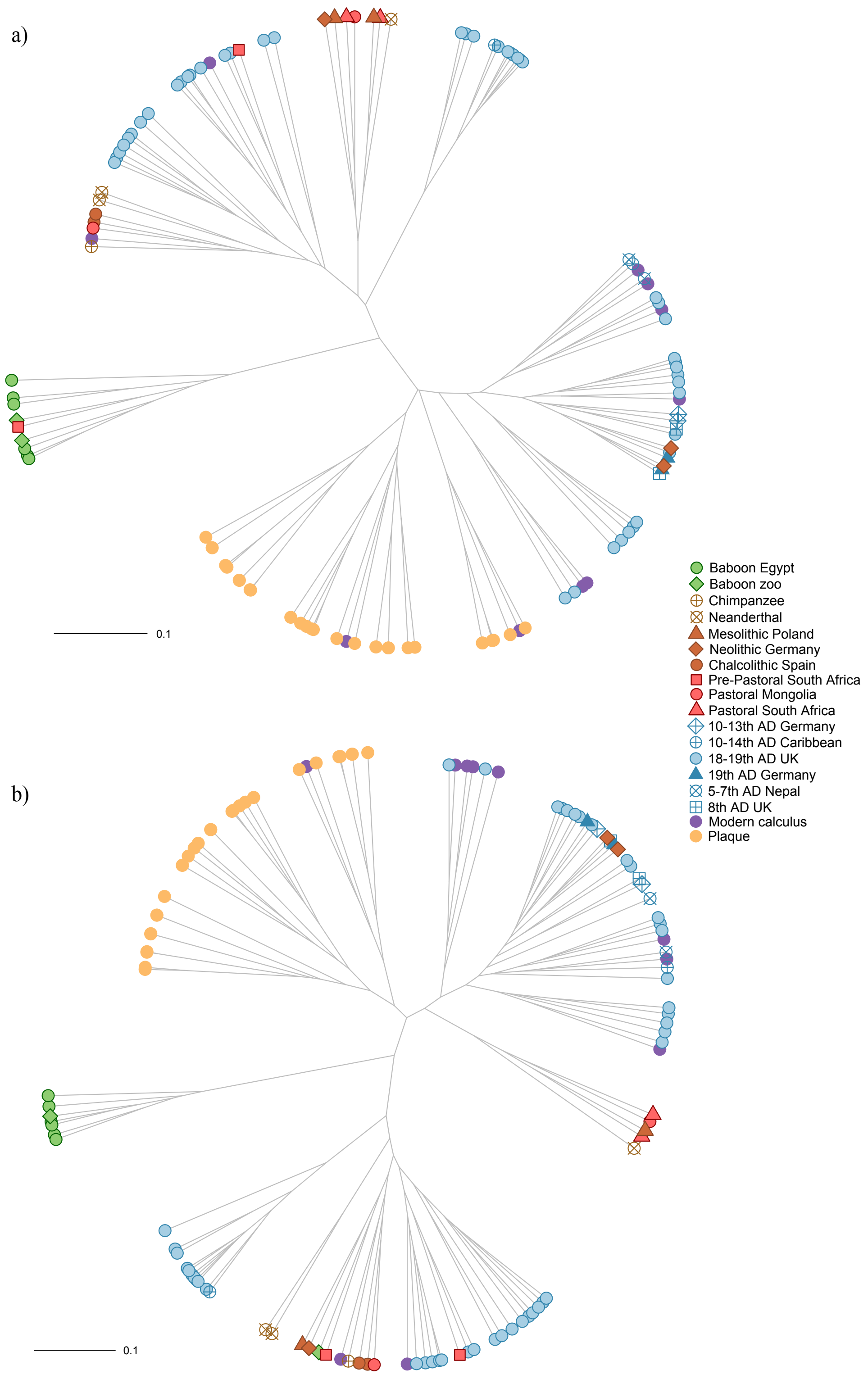

Supplementary figure 3. UPGMA of Bray-Curtis dissimilarities at the genus (a) and species (b) level of oral microbiomes from this study and the literature (Supplementary Table S2).

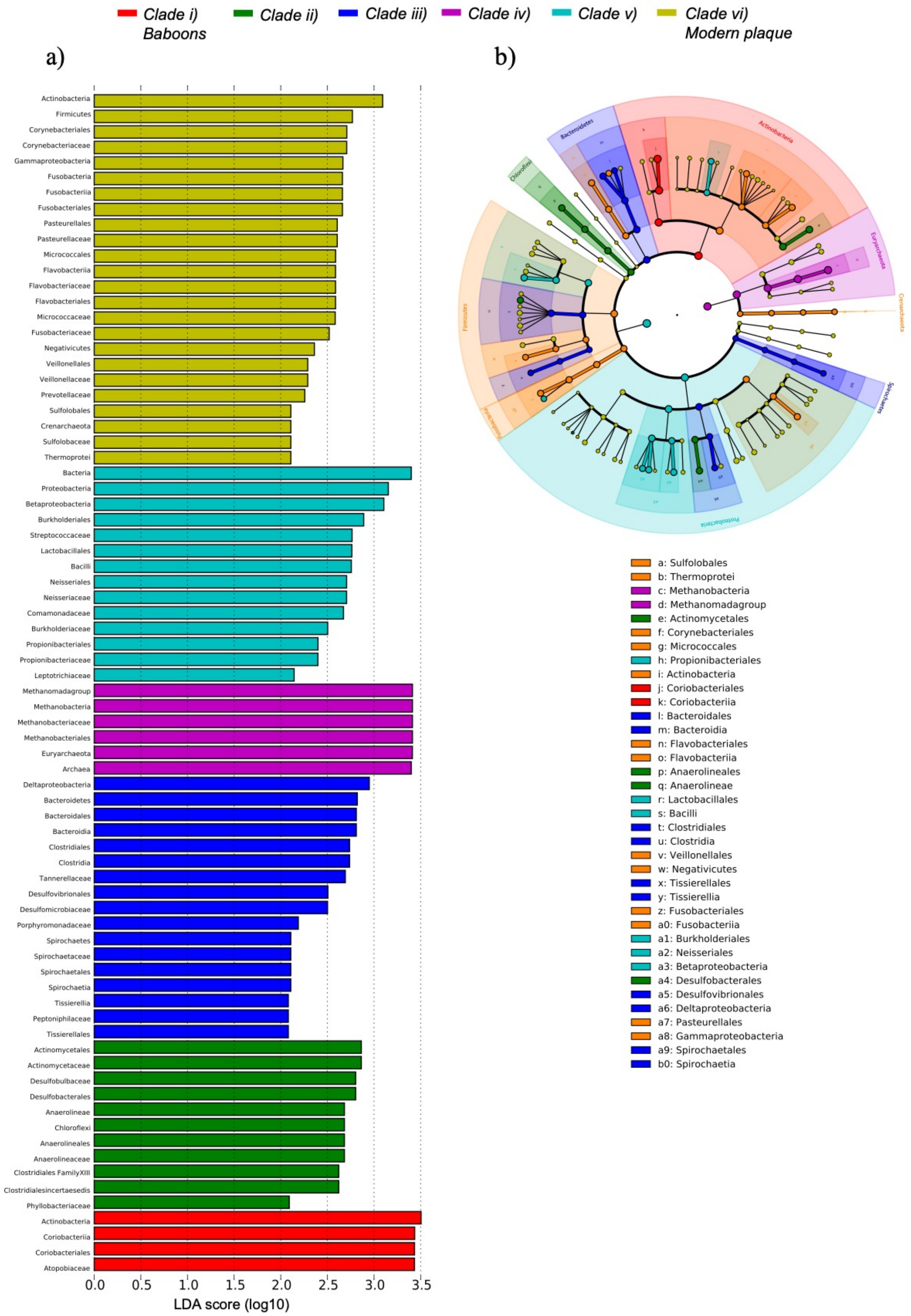

Supplementary figure 4. Discriminating taxa (from phylum to family) identified with LEfSe in the baboon oral microbiome and other oral groups used in this study ranked by effect size (a), and visualized in a cladogram (b).

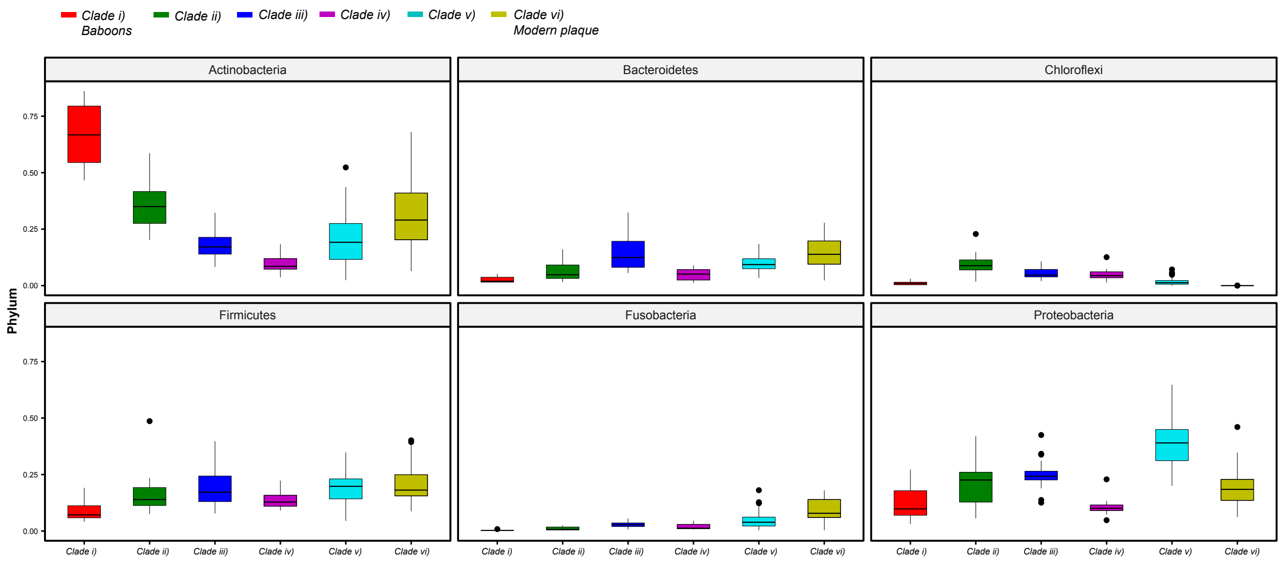

Supplementary figure 5. Box plots of normalized phyla reads abundance across baboons, and other oral microbiomes investigated in this study.

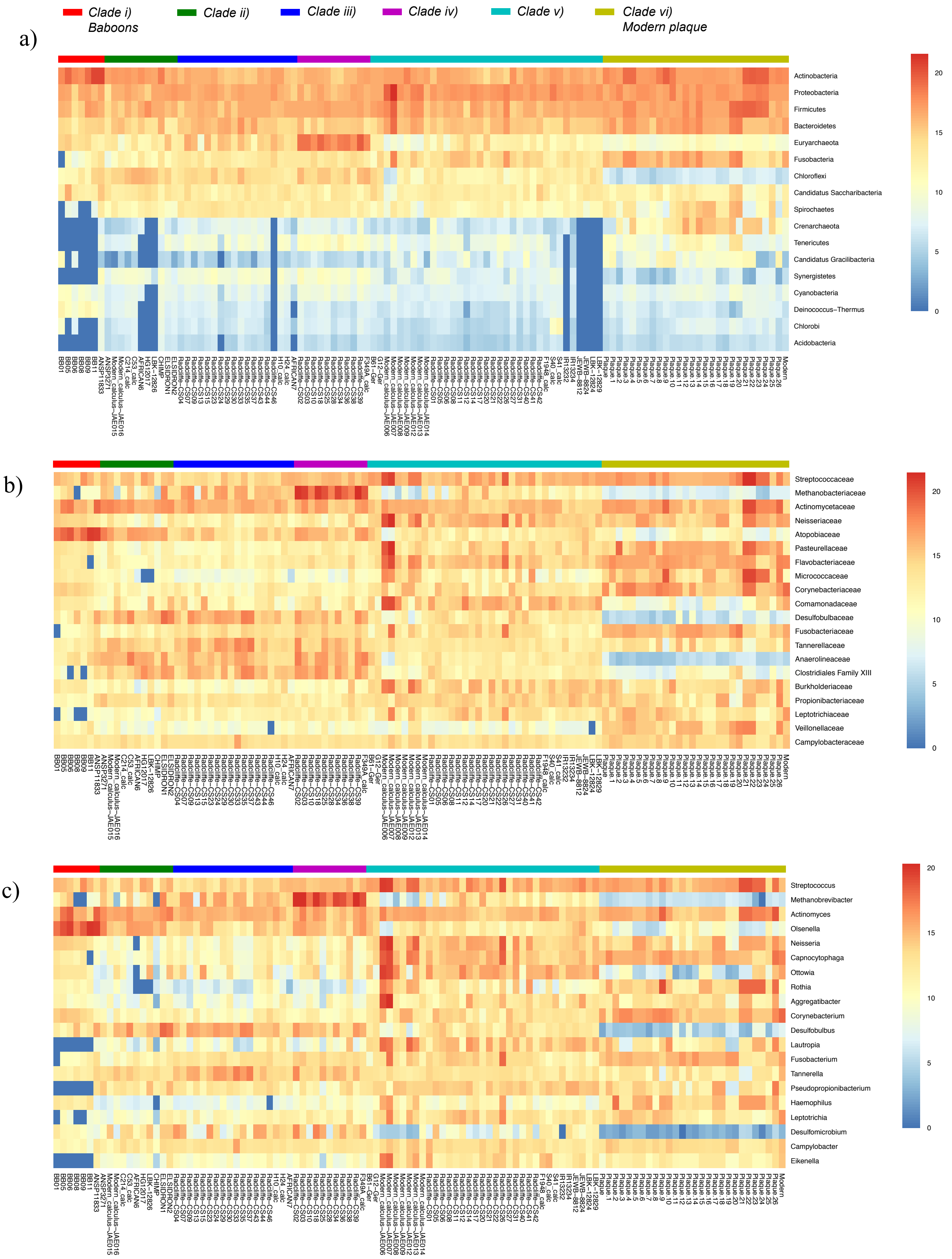

Supplementary figure 6. Heatmaps of normalized phyla (a), families (b) and genera (c) reads abundances in ancient and modern oral microbiomes analysed in this study.

a)

b)

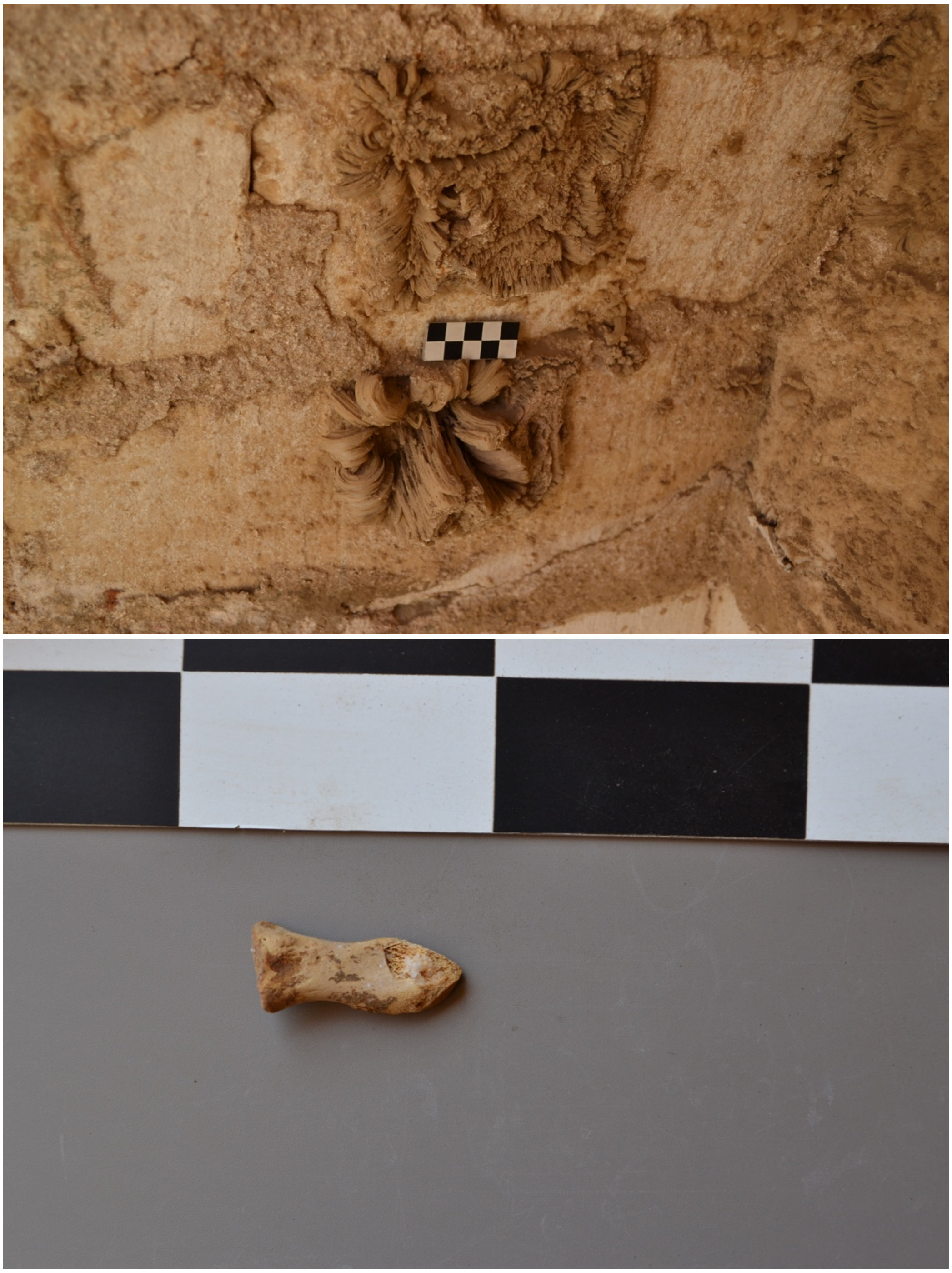

Supplementary figure 7. Example of salt deposit observed in (a) Egyptian burial contexts (Tomb 21 in sector 26 of Oxyrhynchus), and (b) upon archaeological remains (sample of cat ilion from Oxyrhynchus; mummy oxi 13 / s. 26 / UE 26198).
